# Sexual dimorphism in the mast cell transcriptome and the pathophysiological responses to immunological and psychological stress

**DOI:** 10.1186/s13293-016-0113-7

**Published:** 2016-11-22

**Authors:** Emily Mackey, Saravanan Ayyadurai, Calvin S. Pohl, Susan D’ Costa, Yihang Li, Adam J. Moeser

**Affiliations:** 1 Gastrointestinal Stress Biology Laboratory, Michigan State University, East Lansing, MI 48824 USA; 2 Department of Large Animal Clinical Sciences, College of Veterinary Medicine, Michigan State University, East Lansing, MI 48824 USA; 3 Neuroscience Program, Michigan State University, East Lansing, MI 48824 USA; 4 Department of Physiology, Michigan State University, East Lansing, MI 48824 USA; 5 Comparative Biomedical Sciences Program, College of Veterinary Medicine, North Carolina State University, Raleigh, NC 27603 USA; 6 Department of Medicine, University of North Carolina, Chapel Hill, NC 27599 USA

**Keywords:** Stress, Females, Sexual dimorphism, Mast cells, Intestine, Allergy

## Abstract

**Background:**

Biological sex plays a prominent role in the prevalence and severity of a number of important stress-related gastrointestinal and immune-related diseases including IBS and allergy/anaphylaxis. Despite the establishment of sex differences in these diseases, the underlying mechanisms contributing to sex differences remain poorly understood. The objective of this study was to define the role of biological sex on mast cells (MCs), an innate immune cell central to the pathophysiology of many GI and allergic disorders.

**Methods:**

Twelve-week-old C57BL/6 male and female mice were exposed to immunological stress (2 h of IgE-mediated passive systemic anaphylaxis (PSA)) or psychological stress (1 h of restraint stress (RS)) and temperature, clinical scores, serum histamine, and intestinal permeability (for RS) were measured. Primary bone marrow-derived MCs (BMMCs) were harvested from male and female mice and analyzed for MC degranulation, signaling pathways, mediator content, and RNA transcriptome analysis.

**Results:**

Sexually dimorphic responses were observed in both models of PSA and RS and in primary MCs. Compared with male mice, female mice exhibited increased clinical scores, hypothermia, and serum histamine levels in response to PSA and had greater intestinal permeability and serum histamine responses to RS. Primary BMMCs from female mice exhibited increased release of β-hexosaminidase, histamine, tryptase, and TNF-α upon stimulation with IgE/DNP and A23187. Increased mediator release in female BMMCs was not associated with increased upstream phospho-tyrosine signaling pathways or downstream Ca^2+^ mobilization. Instead, increased mediator release in female MCs was associated with markedly increased capacity for synthesis and storage of MC granule-associated immune mediators as determined by MC mediator content and RNA transcriptome analysis.

**Conclusions:**

These results provide a new understanding of sexual dimorphic responses in MCs and have direct implications for stress-related diseases associated with a female predominance and MC hyperactivity including irritable bowel syndrome, allergy, and anaphylaxis.

**Electronic supplementary material:**

The online version of this article (doi:10.1186/s13293-016-0113-7) contains supplementary material, which is available to authorized users.

## Background

Biological sex is a major determinant in the prevalence and severity of many diseases. An increasing number of clinical and epidemiologic studies have demonstrated that the prevalence of allergic disorders, such as urticaria, angioedema, allergic rhinitis, and eczema, is higher in females than males [1–4]. In addition, women are more likely than men to be diagnosed with allergic asthma and develop more severe manifestations of the disease [5, 6]. The incidence of systemic anaphylaxis is higher in females than males, as shown in a number of clinical and epidemiologic studies [3, 7–9]. Sex differences are well-established in gastrointestinal functional disorders such as irritable bowel syndrome (IBS), which is two to four times more prevalent in women than men throughout the world [10]. Despite the known sex differences in disease prevalence and severity, the mechanisms underlying these sex differences remain poorly understood. Furthermore, there is a lack of knowledge regarding the influence of sex in preclinical research [11], and the inclusion of sex as a variable in cell and tissue culture is rare [12]. While both hormonal and non-hormonal factors have been implicated in sexual dimorphism of disease, the reason behind female predominance in these allergic and inflammatory diseases is understudied and largely unknown.

Mast cells (MCs) are tissue-resident innate immune cells that play a critical role in many allergic and inflammatory diseases. A distinguishing feature of MCs is their numerous electron-dense secretory granules found in their cytoplasm that are filled with potent immune mediators such as biogenic amines, proteases, lysosomal enzymes, and cytokines [13]. When MCs are activated, they undergo degranulation and release the contents of the secretory granules into the extracellular space [14]. Released mediators have diverse and profound physiological effects including increases in vascular and epithelial permeability and inflammation, which lead to clinical manifestations of disease [15]. Mast cells have central effector functions in diseases such as allergy and IBS [16, 17]. While there is some evidence that sex-specific differences in these diseases are related to sex hormones, particularly estradiol, to our knowledge, there is no information about sex-specific differences in MCs and their contribution to the disease prevalence or severity. Estrogen can induce MC degranulation [18–24], but there are no studies directly addressing inherent differences that exist between MCs derived from males and females and MC-related disease pathogenesis; therefore, a significant knowledge gap exists regarding sex-specific disease responses with this regard. The objectives of the present study were to investigate the influence of biological sex on MC-mediated pathophysiology in animal models of psychological and immunological stress and to characterize the sexually dimorphic biological responses in primary MC cultures.

## Methods

### Animals

C57BL/6 mice derived from founding colony breeders (The Jackson Laboratory, Bar Harbor, ME) were housed under specific pathogen-free conditions in facilities accredited by the Association for Assessment and Accreditation for Laboratory Care (AAALAC) International. Mice were group-housed with littermates in light- and temperature-controlled and provided ad libitum access to water and a standard commercial rodent chow diet.

### Passive systemic anaphylaxis

Male and female 10–12-week-old C57BL/6 mice (The Jackson Laboratory, Stock No: 000664) were injected intraperitoneally (i.p.) with 10 μg of mouse monoclonal anti-DNP IgE. Twenty-four hours later, mice were challenged with 500 μg of 2,4-dinitrophenyl-human serum albumin (DNP-HSA; i.p.; *n* = 8/sex) or PBS (i.p.; *n* = 4/sex). Rectal temperatures were monitored for 120 min post-DNP injection using a TH-5 Thermalert monitoring thermometer with a rectal probe suitable for mice (Physitemp, Clifton, NJ). Clinical scores were also evaluated for 120 min after DNP injection by a system adapted from Chen et al. [25]. Briefly, the scoring system rated the presence of the following clinical symptoms: scratching, abdominal stretching, rubbing around nose, puffiness in face, pilar erecti, reduced activity, increased respiration rate, labored breathing, inability to move after prodding, inability to right itself, tremors, and death. Each symptom present added 1 point to the clinical score. The scores were recorded by an individual who was blinded to experimental treatments and tallied as a sum total score for each time point. Clinical scores of mice undergoing anaphylaxis were normalized to control mice of respective sex.

Mice were then euthanized by CO_2_ inhalation, and the mesentery of the jejunum was collected and stained with toluidine blue (1%, pH 1) to assess tissue MC degranulation. Five high-power fields (×10 magnification) of mesentery from four mice per group were randomly chosen and MCs were counted in a blinded fashion. For serum histamine measurements, male and female mice (PBS, *n* = 4/sex; DNP-HSA, *n* = 4/sex, repeated in three independent experiments) underwent the same treatment but were sacrificed by CO_2_ after 30 min. Blood was immediately collected by cardiac puncture, and serum was harvested to evaluate histamine concentration by competitive histamine ELISA (Oxford Biomedical Research, Rochester Hills, MI).

### Restraint stress protocol

Female and male mice, approximately 10–12 weeks of age, were subjected to restraint stress (RS) by placing them in individual transparent 50-ml plastic conical tubes, modified with air holes for 15 min (*n* = 6/sex), 30 min (*n* = 6/male, *n* = 12/female), and 1 h (*n* = 5/sex). Control mice (non-stressed) of each sex (*n* = 6) remained in their original home cages for 1 h without food and water to avoid confounding effects of water or feed intake during the 1-h test period. Following RS, stressed and control mice were immediately euthanized by CO_2_ inhalation, and serum was collected for evaluation of histamine concentration by competitive histamine ELISA (Oxford Biomedical Sciences, Rochester Hills, MI), corticosterone concentration by competitive corticosterone ELISA (Immuno-Biological Laboratories, Inc., Minneapolis MN), and estradiol concentration by competitive estradiol ELISA (Calbiotech Inc., El Cajon, CA), which has been demonstrated to parallel the gold standard method of gas chromatography/tandem mass spectrometry [26]. Serum testosterone concentration was measured using a solid-phase competitive chemiluminescent ELISA on the Siemens Immulite 2000 at the Diagnostic Center for Population and Animal Health (Lansing, MI). Ileal mucosa sections were collected from non-stressed mice and stressed mice after 1-h RS for Ussing chamber experiments.

### FD4 intestinal paracellular permeability

Ileum was harvested from each animal immediately following euthanasia and opened lengthwise along the anti-mesenteric border. In oxygenated (95% O_2_, 5% CO_2_) Ringer’s solution (154 Na^+^ mM, 6.3 K^+^ mM, 137 Cl^−^ mM, 0.3 H_2_PO_3_ mM, 1.2 Ca^2+^ mM, 0.7 Mg^2+^ mM, 24 HCO_3_^−^ at pH 7.4) at 37 °C, the seromuscular layer was removed from the tissue by micro-dissection. Mucosal-submucosal sections were then mounted in a 0.3-cm^2^ aperture on Ussing chambers (Physiologic Instruments, Inc., San Diego, CA). The tissue was bathed in Ringer’s solution containing 10 mM glucose (serosal side) that was balanced with 10 mM mannitol on the mucosal side. Bathing solutions were oxygenated (95% O_2_, 5% CO_2_) and maintained at 37 °C. After a 30-min equilibration period on Ussing chambers, 4 kDa fluorescein isothiocyanate dextran (FD4; Sigma, 100 mg/ml) was added to the mucosal bathing reservoir of the Ussing chambers. After a 15-min equilibration period, standards were taken from the serosal side of each chamber and a 60-min flux period was established by taking 0.5-ml samples from the mucosal compartment. The quantity of FD4 was established by measuring the fluorescence in mucosal reservoir fluid samples in a fluorescence plate reader at 540 nm. Data were presented as the rate of FD4 flux in ng FD4.min.cm^2^.

### Derivation of primary BMMCs

Bone marrow cells were isolated from the femurs of 7- to 8-week-old male and female C57BL/6 mice and cultured in complete medium containing RPMI 1640 with L-glutamine, 10% heat-inactivated FBS, non-essential amino acids, HEPES buffer, sodium pyruvate, 100 U/ml penicillin, 100 μg/ml streptomycin, 5 ng/ml murine IL-3, and 5 ng/ml murine stem cell factor (SCF). Cells were grown at 37 °C in 5% CO_2_ for 4 to 8 weeks, at which time >98% of cells in the culture were confirmed as mast cells by toluidine blue staining (1%, pH 1) and FcεR1/c-kit double-positive staining by flow cytometry. The *n* for bone marrow-derived MC (BMMC) experiments was defined by independent flasks.

### pMCs

Peritoneal cells were collected from 8- to 10-week-old C57BL/6 male and female mice by performing peritoneal lavage with 5 ml of Hank’s Balanced Salt Solution (1×) supplemented with EDTA (1 mM) to prevent mast cell degranulation. Peritoneal mast cells (pMCs) were isolated using a 70% Percoll gradient as previously described [27]. Purity of separation was confirmed to be >95% by staining with toluidine blue. Peritoneal mast cells were counted using trypan blue exclusion, and equal numbers of cells were lysed using radioimmunoprecipitation (RIPA) buffer supplemented with phosphatase and protease inhibitors, followed by sonication (Sonic Dismembrator Model 100, Fisher Scientific), for later histamine measurement. Peritoneal mast cells were collected from adult Sprague-Dawley rats by performing peritoneal lavage with 10 ml HBSS (1×) with EDTA (1 mM) followed by a 70% Percoll gradient as previously described [27]. All cells were counted and equal numbers were lysed and sonicated for later histamine measurement.

### Mast cell activation and mediator measurement

BMMCs (2.25 × 10^6^ cells/ml) were sensitized with 1 μg/ml mouse monoclonal anti-DNP IgE overnight and then stimulated with 62 ng/mL DNP-HSA for 1 h, with the exception of the β-hexosaminidase assay which was performed at multiple time points and DNP-HSA concentrations (both from Sigma-Aldrich). BMMCs (2.25 × 10^6^ cells/ml) were also stimulated with 1 μM of the Ca^2+^ ionophore A23187 (Sigma-Aldrich) for 1 h. The presence of β-hexosaminidase in the supernatant and cell lysate was evaluated using the substrate p-nitrophenyl N-acetyl-α-d-glucosaminide. The percentage of β-hexosaminidase release was calculated as a percentage of total β-hexosaminidase content. For other mediators, cell supernatants were collected and kept at −80 °C until later evaluation of mediator release. Unstimulated BMMCs and pMCs were lysed using RIPA buffer, sonicated, and kept at −80 °C until further analysis. Histamine levels in cell supernatants and lysates were quantified using a competitive histamine ELISA (Oxford Biomedical Research, Rochester Hills, MI). Tryptase activity was measured in cell supernatants and lysates, and chymase activity was measured in cell lysates using a colorimetric activity assay with substrates (Z-Lys-SBzl and Succ-AAPF-SBzL, respectively; Bachem, Torrance, CA) specific for cleavage by tryptase or chymase when in the presence of heparin, as previously described [28]. TNF-α levels in supernatants were evaluated by quantitative ELISA (eBioscience). Mediator levels were normalized to unstimulated controls when applicable and corrected to 10^6^ cells.

### Quantitative real-time PCR

RNA was isolated using a RNeasy minikit (QIAGEN) from unstimulated BMMCs and IgE-primed BMMCs stimulated with 62 ng/mL DNP-HSA for 0, 1, 2, and 4 h. cDNA (1 μg) was synthesized using the Maxima First Strand cDNA Synthesis Kit with dsDNase (Thermo Scientific), adhering to manufacturer’s protocol. Samples were prepared according to the TaqMan Gene Expression Master Mix (Applied Biosystems) with the following murine primer/probe sets: Tnf (Mm00443258_m1), Il6 (Mm00446190_m1), Il1β (Mm00434228_m1), Il13 (Mm00434204_m1), Ccl3 (Mm00441259_g1), Esr1 (Mm00441259_g1), Esr2 (Mm00599821_m1), Gper1 (Mm02620446_s1), Pgr (Mm00435628_m1), Ar (Mm00442688_m1), Pgrmc2 (Mm01283155_m1), and Hrpt (Mm00446968_m1). Alternatively, samples were prepared according to LightCycler 480 SYBR 1 Green Master Mix with the following primers: TNF-α forward, 5′-GATCGGTCCCCAAAGGGATG-3′ and TNF-α reverse, 5′-TTGAGATCCATGCCGTTGGC-3′; TPH1 forward, 5′-TCAAAAACTGGCAACGTGCT-3′ and TPH1 reverse, 5′-TACTTCAGTCCAAACGGGCG-3′; RPL4 forward, 5′-GAAGCTCAGTCGGGCTTCTC-3′ and RPL4 reverse, 5′-ATGTCGACGTCGCACTTCAT-3′. Quantitative PCR was performed with the 7500 Fast Real-Time PCR System (Applied Biosystems, Waltham, MA). Relative expression of Tnf and Tph1 in unstimulated BMMCs was calculated based on ΔCt with Rpl4 used as the endogenous control gene. Relative expression of Esr1, Esr2, Gper1, Ar, Pgr, and Pgrmc2 in unstimulated BMMCs was calculated based on ΔCt with Hrpt used as the endogenous control gene. Changes in mRNA levels of Tnf, Il6, Il1β, Il4, Il13, and Ccl3 in IgE-DNP stimulated BMMCs were normalized to Hrpt and relative to time point 0 h.

### Phosphotyrosine expression

IgE-primed BMMCs were stimulated with 62 ng/ml of DNP-BSA for 7 min in complete medium. Cell lysates were then collected as described above and quantified using a BCA reagent assay (Thermo Scientific). Samples were separated by SDS-polyacrylamide gel electrophoresis (Bio-Rad) on a 4–12% XT Bis-Tris Gel (Bio-Rad) and transferred to PVDF membrane (Bio-Rad). Blots were probed with Phospho-Tyrosine Mouse mAb (P-Tyr-100) and visualized with appropriate secondary antibody (both from Cell Signaling Technology). Bands were visualized with ChemiDoc MP imager (Bio-Rad). Densitometric analysis was performed using Image Lab Software (Bio-Rad) and values were normalized with β-actin.

### Ca^2+^ mobilization experiments

BMMCs (1.25 × 10^6^ cells/ml) were suspended in buffer (HBSS with 20 mM HEPES) with Fluo-4 AM (Molecular Probes, Eugene, OR) at 37 °C for 1 h. DNP-HSA (62 ng/ml) was then added to IgE-primed BMMCs, and Ca^2+^ ionophore (1 μM) was added to unprimed BMMCs. Fluo-4 AM fluorescence was monitored over 2 min using a microplate fluorometer (Fluoroskan Ascent FL, Thermo Lab Systems) with excitation at 494 nm and emission at 516 nm. Measurements were normalized to baseline fluorescence to evaluate calcium mobilization in the stimulated condition. Peak fluorescence was calculated as the highest fluorescence value within 2 min of stimulation and corrected with the fluorescence value at time zero.

### Transmission electron microscopy

Peritoneal cells were collected as described above. Peritoneal cells were then pelleted, supernatant was removed, and cells were fixed with Trump’s fixative [29]. Samples were post-fixed for 1 h in 1% osmium tetroxide/0.15 M sodium phosphate buffer, dehydrated through a graded series of ethanols, and embedded in PolyBed 812 epoxy resin (Polysciences, Warrington, PA). Using a diamond knife, 70-nm sections were cut and mounted on 200-mesh copper grids, then stained with 4% aqueous uranyl acetate for 15 min and Reynolds’ lead citrate for 8 min [30]. Samples were observed using a LEO EM910 transmission electron microscope operating at 80 kV (Carl Zeiss SMT, Inc., Peabody, MA) and digital images were acquired with a Gatan Orius SC1000 CCD Digital Camera with Digital Micrograph 3.11.0 (Gatan, Inc., Pleasanton, CA).

### Estrous cycle characterization

Vaginal cytology was performed on adult female mice using a fine tip pipette filled with approximately 10 μL of physiological saline flushed into the vagina. Collected cells were then examined by light microscopy to identify predominant cell types and assign estrus cycle stage (proestrus, estrus, metestrus, diestrus) as previously described [31]. Data from metestrus and diestrus were pooled considering the short duration of metestrus (5–6 h) and plasma estrogen concentrations do not differ at these stages [32]. Female mice were vaginally lavaged daily for at least two consecutive estrous cycles and immediately prior to use in experiments. All females in the current study were regularly cycling. Male mice were handled in a similar manner.

### High throughput RNA sequencing

RNA was extracted from unstimulated BMMCs (1.0 × 10^7^) derived from 2-month-old C57BL/6 male and female mice (*n* = 3/sex) using an RNeasy minikit (QIAGEN). Total RNA was sent to the North Carolina State University Genomic Sciences Lab for library preparation and sequencing. Prior to RNA library preparation and sequencing, RNA quality and concentration were first checked on the Bioanalyzer 2100 with an RNA 6000 Nano Chip (Agilent Technologies Inc., Santa Clara, CA). RIN values were >9.8 for all samples. Poly-A mRNA was purified using the oligo-dT beads provided in the NEBNext Poly(A) mRNA Magnetic Isolation Module (New England BioLabs, Ipswich, MA).

Libraries were prepared using the NEBNext Ultra Directional RNA Library Prep Kit for Illumina and indexed with the NEBNext Mulitplex Oligos for Illumina. Briefly, the Poly-A mRNA was chemically fragmented and primed with random oligos for first-strand synthesis with a heating step of 94 °C for 5 min. First-strand synthesis was performed with an incubation time of 10 min at 25 °C, 50 min at 42 °C, and 15 min at 70 °C. Second-strand synthesis was performed with dUTPs to preserve strand orientation information. The sample was purified, end repaired, and dA-tailed, and NEBNext adaptors were ligated. The ligation reaction was purified according to the protocol for a 450-bp insert, and a 15-cycle PCR amplification was performed, in which an index sequence was incorporated. The PCR product was purified and libraries were checked for quality and concentration on the Bioanalyzer 2100 with a High Sensitivity DNA Chip (Agilent). Libraries were pooled in equal molar amounts and sequenced on the Illumina HiSeq 2500 generating 100-bp SE reads. Each sample generated over 25 million SE reads.

Reads were trimmed of adapter sequences (Trimmomatic 0.32) to remove the TruSeq3-indexed adapter. Reads were aligned to the GRCm38 mouse genome using the TopHat2 program. The CuffDiff program was performed to determine differential expression with a significance cutoff of *P* < 0.05.

### Statistics

Data are represented as the mean ± standard error of the mean (SEM). Statistical analysis using GraphPad Prism 7 software was performed. Two-way ANOVA was used when appropriate, followed by Bonferroni post hoc analysis. Two-way repeated measures ANOVA was used to evaluate rectal temperature and clinical scoring in passive systemic anaphylaxis (PSA) model, followed by the Bonferroni post hoc analysis. One-way ANOVA was used for comparison of more than two groups, followed by Tukey’s multiple comparison test. Unpaired Student’s *t* test or Mann-Whitney *U* test as appropriate was applied to compare differences between two groups. The sample size for each experiment was based upon previous studies and were repeated as indicated in the figure legends. Differences between groups were considered significant at *P* < 0.05.

## Results

### MC responses to immunological stress are sexually dimorphic

**Fig. 1:**
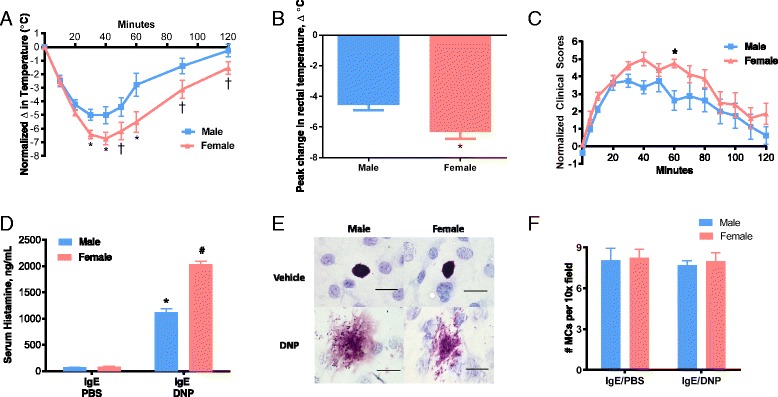
PSA-induced anaphylaxis and serum histamine levels in female and male C57BL/6 mice. **a** Both male and female mice showed a decrease in body temperature after injection with IgE anti-DNP antibody (10 μg/mouse, i.p.), followed 24 h later with vehicle (PBS i.p., *n* = 4/sex) or DNP-HSA (500 μg/mouse, i.p., *n* = 8/sex). Female mice exhibited a trend (*p* < 0.06); Two-way ANOVA on repeated measures) (*p* < 0.05 with individual t-tests at each time point) of lower body temperature than male mice after anaphylaxis challenge along with females demonstrating more severe peak temperature drop (**b**). **c** Over the 120-min PSA challenge, females exhibited a trend of increased clinical scores of anaphylaxis, compared to males, a difference that was statistically significant around the peak of symptoms at 60 min. **d** Serum levels of histamine, as measured by ELISA 30 min after DNP (values represent results from one individual experiment with *n* = 4 animals/sex; results were replicated in three independent experiments) showed that both male and female mice had an increase in histamine, and the increase was greater for females. **e** Representative photomicrographs of intestinal mesentery from male and female mice stained with toluidine blue showing increased degranulation in DNP-treated animals; original magnification, ×100, *bar* = 20 μM. **f** The number of mast cells was similar in female and male mice. Mast cells were counted in five randomly chosen fields per mouse. Values represent mean ± SE. †*P* < 0.10, *P < 0.05, ^#^ *P* < 0.0001

To investigate whether female and male mice exhibited differences in MC activation, we used a well-characterized MC-dependent model of PSA. Adult male and female mice were sensitized with mouse anti-DNP IgE monoclonal antibody, via i.p. injection and then challenged 24 h later with DNP to induce anaphylaxis. Body temperature, clinical scores, and serum histamine were measured as direct and indirect markers of MC activity. Compared with male mice, female mice exhibited a trend for more severe hypothermia when measured over the 120-min post-PSA induction (*F*_(1,14)_ = 4.118, *p* = 0.0619 for sex effect; *F*_(8.112)_ = 116.5, *p* < 0.0001 for time effect, interaction: *F*(_8,112)_ = 5.05, *p* < 0.0001) while the peak drop in body temperature was significantly greater in females (Δ body temp = −4.53 ± 0.38 °C vs. −6.29 ± 0.45 °C in male and female mice, respectively; *p* < 0.05) (Fig. 1a, b). There was also a trend for female mice to exhibit more severe clinical symptoms of anaphylaxis, as indicated by increased clinical scores compared with male mice (*F*_(1,14)_ = 3.239, *p* = 0.0935 for sex effect, *F*_(13,182)_ = 29.11, *p* < 0.0001 for time effect; Fig. 1c). Serum levels of the MC granule mediator histamine, which is responsible for increased vascular permeability and symptoms of anaphylaxis including hyperemia, swelling, and hypothermia [33] were greater (by 1.8-fold) in female mice (2032 ± 64 ng/mL) compared with male mice (1121 ± 67 ng/mL) (*F*_(1,12)_ = 96.8, *p* < 0.0001 for sex effect, *F*_(1,12)_ = 1032, *p* < 0.0001 for treatment effect, interaction: F_(1,12)_ = 93.7, *p* < 0.0001; Fig. 1d). To confirm tissue MC degranulation, we evaluated tissue MCs via toluidine blue staining in intestinal mesentery windows. While extensive degranulation was observed in response to PSA, there was no noticeable difference in MC activation patterns, such as extent of degranulation or percentage of MCs degranulated, between males and females (Fig. 1e). In addition, there were comparable numbers of MCs present in the intestinal mesentery of male and female mice, thus suggesting that heightened anaphylactic responses were not a result of increased MC numbers in female mice (Fig. 1f).

### MC responses to psychological stress are sexually dimorphic

**Fig. 2:**
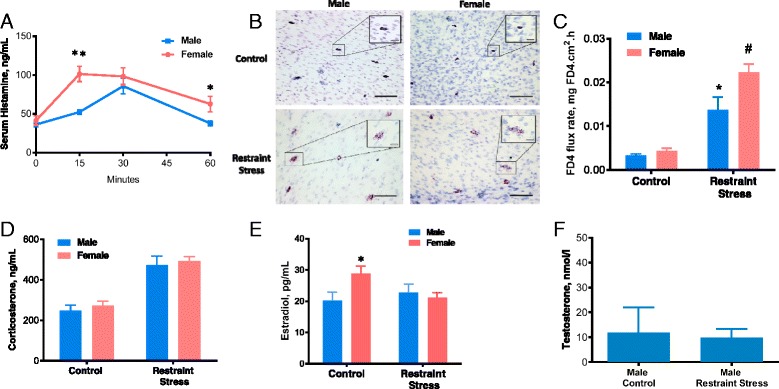
Psychological RS-induced intestinal permeability and serum histamine levels in female and male C57BL/6 mice. **a** After experiencing 15 min (*n* = 6/sex), 30 min (*n* = 6/male, *n* = 12/female), and 1 h (*n* = 5/sex) of restraint stress (RS), females exhibited a statistically significant increase in serum histamine (t-test; *p*<0.05), as measured by ELISA, compared to males. **b** Representative photomicrographs of intestinal mesentery from male and female mice stained with toluidine blue showing increased degranulation in RS animals; original magnification, ×20, *bar* = 100 μM, inset; ×100, *bar* = 20 μM. **c** Distal ileum harvested after RS demonstrated increased permeability for both females and males, compared to baseline (*n* = 3–4/sex). After RS, female intestinal permeability was 0.0223 ng cm^2^/h and male intestinal permeability was 0.0137 ng cm^2^/h. **d** Serum corticosterone increased similarly in male and female mice after 30 min RS (*n* = 6/male, *n* = 12/female). **e** Serum estradiol decreased in female mice after 30 min of RS (*n* = 6/male, *n* = 12/female). **f** Serum testosterone levels were similar in males before and after RS (*n* = 6). Values represent mean ± SE, *P < 0.05, **P<0.01 vs. other treatments

To determine if the heightened MC responses and pathophysiology exhibited in female mice was specific to the IgE-dependent PSA model, we also measured MC responses (through serum histamine levels) between males and females in a non-IgE-dependent model of psychological RS. Female and male mice were subjected to short periods (15 min, 30 min, 1 h) of RS, after which serum histamine was measured. Restraint stress induced an elevation in serum histamine that was greater in female mice as compared to male mice, most notably at the 15-min time point (101.6 ± 9.906 vs. 52.39 ± 3.086, *F*_(1,44)_ = 10.62, *p* =0.0022 for sex effect, *F*_(3,44)_ = 13.1, *p* < 0.0001 for time effect) (Fig. 2a). Toluidine blue staining of intestinal mesentery confirmed that RS led to MC degranulation in both male and female mice (Fig. 2b). Intestinal permeability, a MC-dependent pathophysiologic response to RS, was measured on Ussing chambers following 1 h of RS and revealed similar findings to serum histamine in that female mice exhibited greater RS-induced intestinal permeability compared with males (*F*_(1,9)_ = 6.563, *p* = 0.0306 for sex effect, *F*_(1,9)_ = 57.03, *p* < 0.0001 for stress effect; Fig. 2c). Corticosterone, the main glucocorticoid involved in stress responses in rodents, was similarly elevated in male and female mice after RS demonstrating a similar activation of the hypothalamic-pituitary-adrenal axis (HPA) response induced by RS in both sexes (*F*_(1,30)_ = 61.5, *p* < 0.0001 for stress effect; Fig. 2d). Because previous studies have shown that stress can alter the secretion of sex steroids by interfering with the hypothalamic pituitary-gonadal axis, we determined whether serum concentrations of estradiol or testosterone differed between male and female mice in response to RS. As expected, serum estradiol was higher in female mice compared with male mice under control, non-stressed conditions (Fig. 2e). However, restraint stress reduced serum estradiol in female mice which was in line with previously published work [34]. However, serum estradiol levels remained unaltered when measured at 1 h after RS in male mice (*F*_(1,28)_ = 4.629, *p* = 0.0402 for interaction; Fig. 2e). Also comparable to previous studies [35], testosterone levels were not reduced in males in response to restraint stress (Fig. 2f). Serum testosterone measurements were below the limit of detection for female mice in our experiments (data not shown). Together, results from the PSA and RS models indicate that female mice display heightened MC activation in response to diverse immunological and psychological stressors which was associated with more severe MC-associated clinical and tissue pathophysiology.

### Female BMMCs exhibit increased degranulation and granule mediator release

**Fig. 3:**
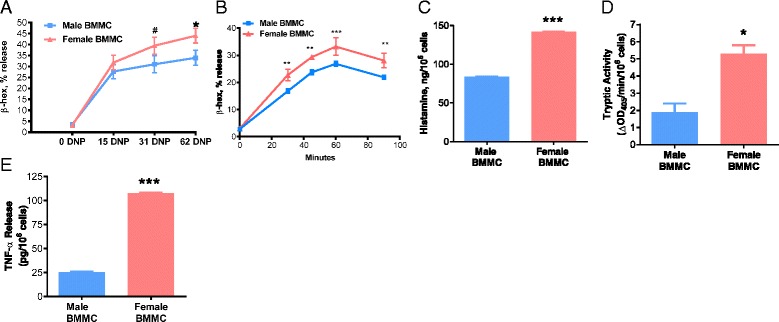
Female BMMCs release more preformed mediators in response to IgE-mediated degranulation. BMMCs were sensitized with anti-DNP IgE (1 μg/mL) overnight and later stimulated with DNP-HSA. **a** Male and female BMMCs were stimulated with 0, 15, 31, and 62 ng/mL of DNP-HSA for 1 h and female BMMCs exhibited elevated β-hexosaminidase release at all DNP concentrations (*P* < 0.05; *n* = 6). **b** Compared with male BMMCs, female BMMCs had an increased release of β-hexosaminidase after 30, 45, 60, and 90 min of DNP-HSA stimulus (62 ng/mL). **c** Female BMMCs released 141.9 ng/10^6^ cells of histamine into supernatant and male BMMCs released 83.7 ng/10^6^ cells of histamine after 1 h DNP stimulus (62 ng/mL) (*P* < 0.001; *n* = 5). **d** Tryptic activity from female BMMCs increased to 5.3 after degranulation, and tryptic activity from male BMMCs was 1.9 after degranulation with 1 h DNP stimulus (62 ng/mL) (*P* < 0.05; *n* = 6). **e** Female BMMCs released 107.4 pg of TNF-α into supernatant and male BMMCs released 25.5 pg after 1 h DNP stimulus (62 ng/mL) (*P* < 0.001; *n* = 5). All mediator release was normalized to unstimulated cells of respective sex. Values represent mean ± SE. ^#^ *P* = 0.10 *P < 0.05, **P<0.01 ***P < 0.001 vs. males

Our in vivo experiments in PSA and RS models demonstrated that females released greater amounts of histamine and exhibited heightened MC-associated pathophysiology. To gain further insight into this sexually dimorphic response, we determined whether male and female MCs exhibited different functional and (or) morphological characteristics, utilizing primary bone marrow-derived mast cells (BMMCs) derived from adult male and female mice. BMMCs were sensitized overnight with DNP-specific IgE (1 μg/mL), followed by DNP-HSA to induce FcεR1 cross-linking and degranulation after which MC granule mediators were measured in the supernatants. Compared with IgE/DNP-stimulated BMMCs derived from males, BMMCs derived from females exhibited a greater release of β-hexosaminidase (a MC granule marker) at multiple time points of stimulus (Fig. 3a; *F*_(1,17)_ = 59.32, *p* < 0.0001 for sex effect, *F*_(4,17)_ = 223.1, *p* < 0.0001 for time effect, interaction: *F*_(4,17)_ = 3.23, *p* = 0.0383; Fig. 3a) and at multiple concentrations of IgE-DNP (Fig. 3b). Female BMMCs also released more histamine (by 1.7-fold), tryptase (by 2.8-fold), and TNF-α (by 4.2-fold) in response to IgE/DNP compared with male BMMCs (Fig. 3c–e). Spontaneous degranulation in unstimulated conditions was not different between male and female BMMCs with regard to any mediators except histamine where female BMMCs had slightly higher spontaneous release in unstimulated conditions (Additional file 1: Figure S1). All stimulated mediator release was corrected with unstimulated controls. Therefore, differences in spontaneous degranulation were not the cause of the increased mediator release found in female BMMCs. Also, there were no noticeable difference in the morphologic appearance between unstimulated male and female BMMCs upon visualization of toluidine blue-stained slides of BMMCs(Additional file 1: Figure S1). Despite an expected upregulation of mRNA expression for multiple cytokines following IgE/DNP stimulation in BMMCs, there were no differences between male and female BMMCs, with the exception of moderately increased expression of IL-1β in male-stimulated BMMCs (*F*_(1,12)_ = 5.255, *p* = 0.0408 for effect, *F*_(2,12)_ = 15.31, *p* = 0.0005 for time effect; Additional file 2: Figure S2A-F).

### Female and male BMMCs exhibit similar induction of FcεRI-mediated phosphotyrosine signaling and intracellular Ca^2+^ mobilization

**Fig. 4:**
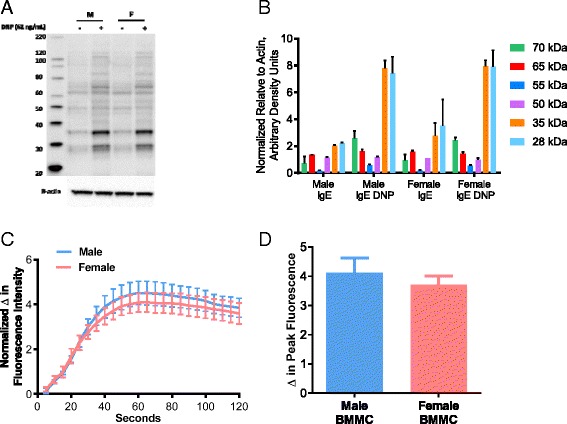
FcεRI-mediated p-tyrosine expression and Ca^2+^ mobilization in female and male murine BMMCs. After sensitization with anti-DNP IgE, BMMCs were stimulated with DNP-HSA (62 ng/ml) for 7 min. Results from female and male BMMCs showed no differences in **a** tyrosine phosphorylation of proteins after FcεRI engagement, **b** quantification of tyrosine-phosphorylated proteins (*n* = 4), or Ca^2+^ influx as indicated by Ca^2+^ tracings (**c**) and quantified as the **d** change in peak fluorescence (*n* = 3). Values represent mean ± SE

To determine the mechanisms that led to increased MC degranulation responses in female MCs, we performed Western blotting to measure FcεR1-mediated tyrosine phosphorylation signaling, a critical upstream signaling event in IgE-mediated MC activation [14], in female and male BMMCs. As expected, IgE-FcεR1 cross-linking induced a robust tyrosine phosphorylation of numerous proteins that were increased in response to IgE-DNP treatment. However, P-tyrosine phosphorylation patterns were similar between female and male BMMCs (Fig. 4a, b). We next investigated whether differences existed in downstream MC degranulation signaling events. Specifically, we compared FcεRI-mediated intracellular Ca^2+^ mobilization responses in male and female BMMCs. Ca^2+^ release from intracellular endoplasmic reticulum stores and subsequent extracellular Ca^2+^ entry through store-operated Ca^2+^ entry (SOCE) channels are major downstream signaling events required for MC granule fusion with the plasma membrane and granule exocytosis [36]. Similar to P-tyrosine data, IgE/DNP induced a robust increase in intracellular Ca^2+^; however, no differences were observed between male and female BMMCs (Fig. 4c, d). Together, these data suggest that heightened MC degranulation responses in female MCs were not due to differences in upstream receptor-mediated signaling or downstream Ca^2+^ mobilization mechanisms.

### Female MCs contain more granule-associated mediators

**Fig. 5:**
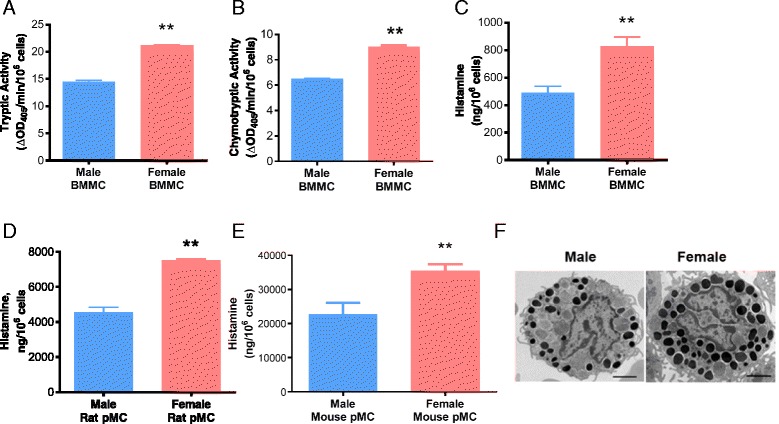
Female mast cells contain more granule-associated mediators than male mast cells. **a** The total tryptic activity of unstimulated female BMMCs was higher than that of male BMMCs (21.2 vs. 14.5; *P* < 0.01; *n* = 5). **b** The total chymase activity of unstimulated female BMMCs was higher than that of male BMMCs (9.0 vs. 6.4; *P* < 0.01; *n* = 3). **c** The total histamine content of unstimulated female BMMCs was higher than that of male BMMCs (827.7 vs. 487.8; *P* < 0.05; *n* = 5). **d** The total histamine content of female mouse pMCs was higher than that of male pMCs (35,489 vs. 22,673; *P* < 0.01; *n* = 7/male, *n* = 11/female). **e** The total histamine content of pMCs from female rats was higher than that of male rat pMCs (71,806 vs. 43,167; *P* < 0.001 *n* = 3). **f** Representative images of peritoneal mast cells recovered and pooled from five, 8-week-old male and female C57BL/6 mice and analyzed by TEM. *Scale bar* = 2 μm. All values were normalized to cell number. Values represent mean ± SE. *P < 0.05, **P < 0.01, ***P < 0.001 vs. males

Given the lack of significant differences in MC signaling pathways described above, we next reasoned that the increased mediator release in female MCs could be due to increased MC granule mediators. Measurement of MC granule mediators in unstimulated cell pellets indeed revealed that female BMMCs contained significantly higher concentrations of tryptase, chymase, and histamine, compared with male BMMCs, (Fig. a–c). To confirm that increased MC granule mediator content in female MCs was not restricted to in vitro cultured BMMCs, we isolated tissue resident MCs from the peritoneal cavity of male and female mice and measured the cellular histamine content. Consistent with the BMMC results, female pMCs contained significantly greater histamine content compared with male pMCs (Fig. 5d). The increased histamine content in female MCs was also confirmed in rat pMCs (Fig. 5e), demonstrating that increased MC mediator content in females was not specific to mice. To further characterize the differences in female and male MCs, we evaluated mouse pMCs at the ultrastructural level using TEM [26]. In agreement with MC mediator data, MC granules from female pMCs visually had increased density compared with male pMCs (Fig. 5f). Together, these data demonstrate that MCs from females contain more granule-associated immune mediators compared with male MCs.

**Fig. 6:**
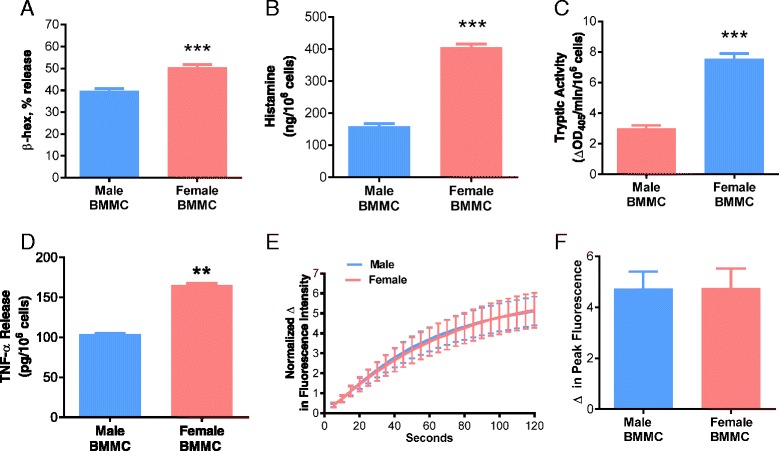
A23187-induced degranulation and Ca^2+^ mobilization in female and male murine BMMCs. BMMCs were stimulated for 1 h with the Ca^2+^ ionophore, A23187 (1 μM). **a** BMMCs from females showed a 50.6% increase in β-hexosaminidase release and male BMMCs showed a 39.9% increase, a difference that was significant (*P* < 0.001; *n* = 6). **b** Female BMMCs released 406.1 ng/10^6^ cells of histamine into supernatant and male BMMCs released 159.3 ng/10^6^ cells of histamine (*P* < 0.001; *n* = 5). **c** Tryptic activity (ΔOD405/min/10^6^ cells) from female BMMCs increased to 7.6 after degranulation, and tryptic activity from male BMMCs was 3.2 after degranulation (*P* < 0.001; *n* = 4). **d** Female BMMCs released 165.7 pg of TNF-α into supernatant and male BMMCs released 103.4 pg (*P* < 0.01; *n* = 4). All mediator release was normalized to unstimulated cells of respective sex. **e** Female and male BMMCs exhibited similar Ca^2+^ influx as measured by **e** Ca^2+^ tracings and quantified as the **f** change in peak fluorescence after stimulation with A23187 (1 μM). Values represent mean ± SE. **P < 0.01, ***P < 0.001 vs. males

As an additional functional experiment to confirm that the increased granule mediator content in female mice was contributing to heightened mediator release during degranulation, we measured MC degranulation and Ca^2+^ mobilization in BMMCs in response to the Ca^2+^ ionophore A23187, which acts downstream of receptor activation to trigger Ca^2+^ influx and MC degranulation. Similar to IgE/DNP-stimulation responses, female BMMCs released greater amounts of β-hexosaminidase, histamine, tryptase, and TNF-α in response to A23187 than male BMMCs. Also in line with IgE/DNP stimulation, there were no differences in Ca^2+^ mobilization responses between male and female BMMCs (Fig. 6a–f).

### Sexual dimorphic MC responses are not influenced by estrous cycle

**Fig. 7:**
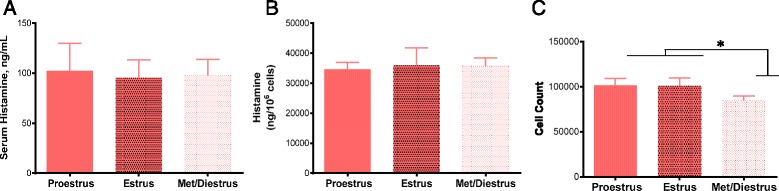
Influence of the estrous cycle on serum histamine response and tissue MC histamine content and number. **a** After experiencing 30 min of RS, serum histamine levels were similar between female mice in all stages of the estrous cycle. **b** The total histamine content of mouse female pMCs was similar at each stage of the estrous cycle. **c** During proestrus and estrus, female mice had higher numbers of pMCs than male mice. Values represent mean ± SE. *P < 0.05 vs. males

The estrous cycle, characterized by fluctuations of ovarian steroid hormones, has been shown to influence MC density and phenotype in multiple rodent tissues [37, 38]. Therefore, we sought to evaluate whether the estrous cycle was influencing the sexually dimorphic MC pathophysiology and phenotype demonstrated in the present study. Serum histamine levels following RS did not differ between female mice in different stages of the estrous cycle (Fig. 7a). Similarly, no differences were found in histamine content of pMCs in female mice with regard to the estrous cycle (Fig. 7b). Interestingly, a mildly increased number of pMCs was found in female mice in proestrus and estrus in comparison to Met/Diestrus, similar to findings previously discovered in rat mammary gland tissue (*F*_(3,14)_ = 4.303, *p* = 0.0239; Fig. 7c) [39].

Sex steroid hormones, in particular estradiol, have been previously demonstrated to induce mild degranulation in BMMCs [19, 21]. Therefore, we evaluated whether sex steroid receptors were differentially expressed on male and female BMMCs, providing another potential mechanism for increased MC degranulation responses in female BMMCS. These studies revealed that female BMMCs had higher mRNA expression of *Esr1* and *Ar* correlating to estrogen receptor α and androgen receptor, respectively (Additional file 3: Figure S3A-F).

### Transcriptomic analysis in BMMCs reveals sexual dimorphism

**Fig. 8:**
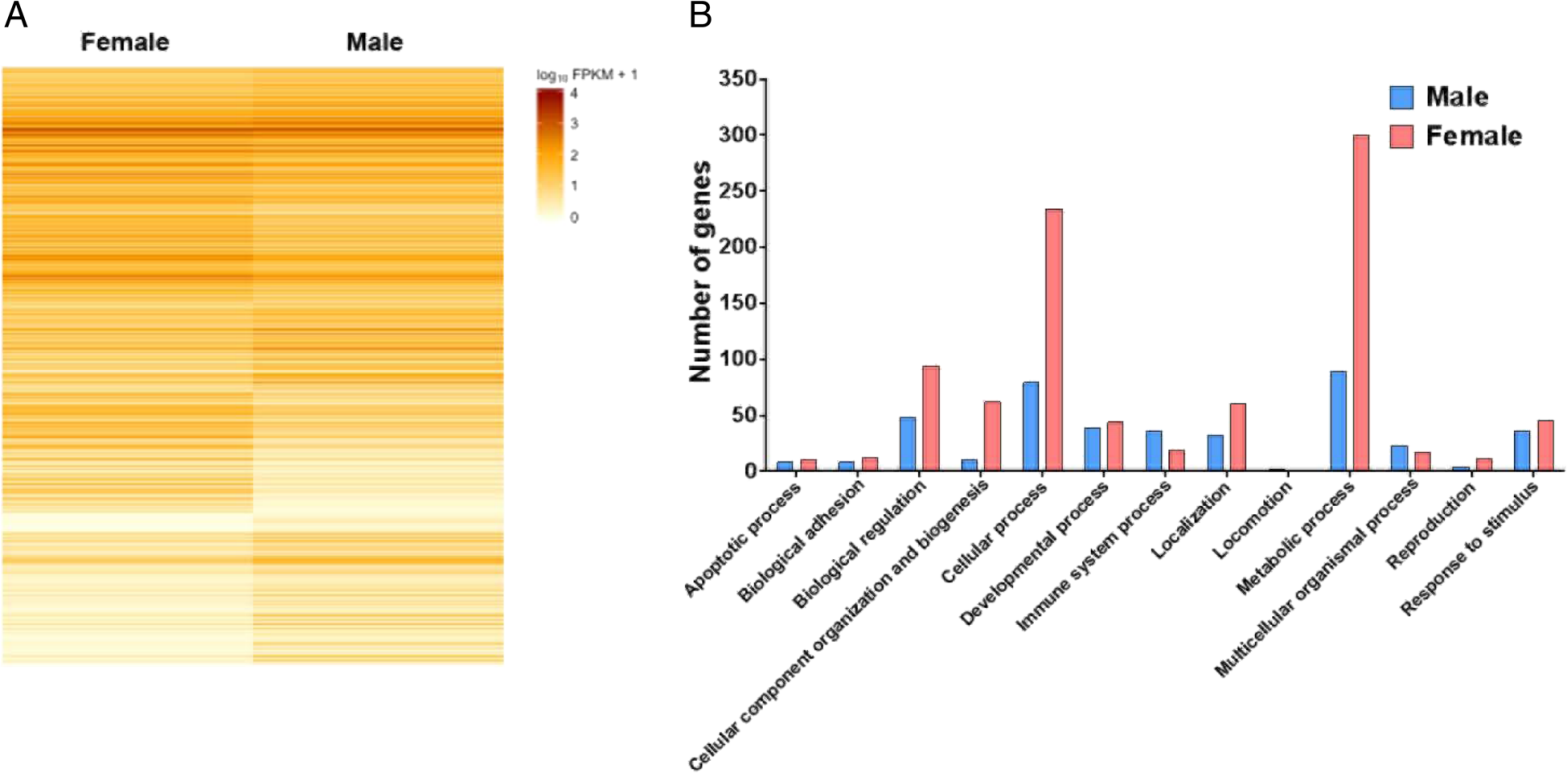
Sexually dimorphic gene expression in BMMCs from C57BL/6 mice. **a** A total of 8233 genes were found to be differentially expressed between 6-week-old male and female BMMCs using RNA-sequencing technology (Illumina HiSeq 2500) (*n* = 3). **b** There were many genes that code for proteins involved in cellular processes and metabolic processes that were upregulated in females compared to males

To gain a more fundamental understanding of why MCs from females contained increased MC granule mediators, we profiled the gene expression patterns in MCs from males and females using RNA sequencing technology (Illumina HiSeq 2500) with RNA extracted from unstimulated BMMCs (1.0 × 10^7^ cells). Remarkably, a total of 8233 genes were expressed differentially between male and female BMMCs (Fig. 8a). This finding demonstrates that, while male and female cells are generally assumed to have very similar autosomal genomes, the expression of the genome can vary immensely between the sexes. The *Xist* gene, which is exclusively expressed from the inactive X chromosome in females, had the greatest differential expression in females (9.9-fold) [40]. Similarly, two Y chromosome genes, *Eif2s3y* and *Kdm5d*, had the greatest differential expression in males (7.6-fold and 8.6-fold, respectively). These results validate RNA sequencing to determine sex-specific differences in BMMCs.

We next evaluated gene ontology for biological processes between male and female BMMCs using Protein ANalysis THrough Evolutionary Relationships software (PANTHER), focusing on the genes with >2-fold difference in expression (Fig. 8b). Interestingly, MCs from females had more than twice as many upregulated genes (552 vs. 250 genes) involved in biological processes compared with MCs from males. Of particular interest to our current findings, the metabolic processes category had 300 genes upregulated in females vs. 89 in males, and within this category, biosynthetic processes had 35 genes upregulated in females and only 1 in males. Female BMMCs also exhibited higher expression of genes in cellular processes, especially genes involved in the cell cycle, as well as higher expression of genes in cellular component organization and biogenesis than male BMMCs.

Considering our findings that female MCs contain more intracellular granule mediators than male MCs, we next investigated whether genes for immune mediators known to be stored in preformed granules of MCs were upregulated in females (Table 1). We found that *Tnf* gene expression was 3.48-fold higher in female BMMCs. Mast cell protease 1, which is a β-chymase often associated with mucosal MCs and abundantly found in BMMCs, was found to be 2.70-fold higher in female BMMCs [41]. *Mcpt2*, *mcpt4*, and *mcpt8* were also found to have higher gene expression in female BMMCs than male BMMCs, as well as the gene for the alpha subunit of the lysosomal hydrolase β-hexosaminidase, *Hexa*.

**Table 1:** Expression of genes in female BMMCs relative to male BMMCs, involved in forming immune mediators stored in mast cell granules

Gene name	Gene symbol	Comments	Statistics	
			Log_2_ fold change	*q* value
Tumor necrosis factor	*Tnf*	Proinflammatory cytokine stored in preformed mast cell granules (Gordon & Galli 1990 [81])	3.48	0.00016
Mast cell protease 1	*Mcpt1*	B-chymase involved in maintaining granule structure (Wastling 1998 [82]) and inflammation at mucosal sites [41]	2.70	0.015
Mast cell protease 2	*Mcpt2*	Enzymatically inactive chymase without known function (Caughley 2011 [83])	0.64	0.00016
Mast cell protease 4	*Mcpt4*	Functional equivalent to human mast cell chymase (Caughley 2015 [43])	0.39	0.037
Mast cell protease 8	*Mcpt8*	Serine protease more closely related to granzymes and cathepsin G than chymases (Lutzelschwab 1998 [84])	1.71	0.00016
Tryptase beta 2	*Tpsb2*	Serine protease important for pathology in a variety of inflammatory conditions (Payne 2004 [85])	−1.35	0.00016
Cathepsin B	*Ctsb*	Cysteine protease that processes protryptase to mature tryptase (Le 2011a [86])	0.31	0.015
Cathepsin C	*Ctsc*	Cysteine protease that is an upstream activator of tryptases, chymases, and cathepsin G (Caughley 2015 [43])	0.99	0.00016
Cathepsin G	*Ctsg*	Serine protease with tryptic and chymotryptic activity (Caughley 2007 [87])	1.16	0.00016
Cathepsin H	*Ctsh*	Cysteine protease that may be involved in tumor progression (Schweiger 2004 [88])	1.28	0.0099
Cathepsin K	*Ctsk*	Cysteine protease that degrades type 1 collagen (Bone 2004 [89])	1.71	0.00016
Cathepsin L	*Ctsl*	Cysteine protease that processes protryptase to mature tryptase (Le 2011a [86])	1.09	0.00016
Cathepsin S	*Ctss*	Cysteine protease linked to inflammatory processes such as atherosclerosis, asthma, and atopic dermatitis [20]	1.28	0.00016
Cathepsin Z	*Ctsz*	Cysteine protease involved in enhanced metastasis in various cancers (Wang 2011 [90])	1.02	0.00016
Cathepsin E	*Ctse*	Aspartic protease that processes procarboxypeptidase (Henningsson 2005 [91])	−2.05	0.00016
Cathepsin F	*Ctsf*	Cysteine protease most notably found in neural tissue (Tang 2006 [92])	−2.46	0.00016
Cathepsin O	*Ctso*	Cysteine protease with endoprotease activity (Zhang 2015 [93])	−1.09	0.00016
B-hexosaminidase subunit alpha	*Hexa*	Alpha subunit of the lysosomal hydrolase B-hexosaminidase, an important enzyme in defense against pathogens (Fukuishi 2014 [94])	0.32	0.0038
Phosphoribosyl pyrophosphate synthetase 1	*Prps1*	Key enzyme involved in histidine and tryptophan synthesis [68, 44]	2.45	0.00016
Phosphoribosyl pyrophosphate synthetase 2	*Prps2*	Key enzyme involved in histidine and tryptophan synthesis [68, 44]	0.54	0.00074
Tryptophan hydroxylase 1	*Tph1*	Key enzyme in the synthesis of serotonin from tryptophan (Nowak 2012 [49])	0.55	0.0011

*Q* values <0.05 are considered significant

Surprisingly, tryptase beta 2 was found to be downregulated in female BMMCs, which does not correlate with our finding that female mast cells have increased tryptic activity. However, while protein concentration generally correlates with the abundance of the corresponding mRNAs, regulation of post-transcription, translation, and protein degradation determines functional protein abundance [42]. Gene expression for the most abundant MC-associated cathepsins (G, C, L, and S [43]) were all upregulated in females. Gene expression of key enzymes involved in the first steps of synthesizing histidine and tryptophan, phosphoribosyl pyrophosphate synthetase 1 and 2, were found to be upregulated in female BMMCs as well as a gene for the enzyme tryptophan hydroxylase, which converts tryptophan to serotonin [44–46]. Together, these data demonstrate a higher expression in females than males of numerous genes involved in forming immune mediators that are stored in preformed secretory granules in BMMCs and further support our findings in vitro.

We also evaluated whether genes involved in MC granule biogenesis and maturation were increased in female MCs because of the increased mediator content found in the granules of female MCs (Table 2). *Srm*, *Odc1*, and *Sms*, which are genes involved in polyamine synthesis and therefore granule maturation, were upregulated in female BMMCs [47]. Another gene involved in granule maturation that was upregulated in female mast cells was *Bhlha15*, which is also referred to as *Mist1*. Rab5 is a small GTPase known to play an important role in granule biogenesis in mast cells, and the gene *Rab5c* was upregulated in females [48]. Genes *Ap1m1* and *M6pr*, which code for proteins that sort mediators into vesicles/granules, were upregulated in female BMMCs [49, 50]*.* Many genes in the *Atp6v* family, which correspond to proteins that help acidify granule lumens, were also upregulated in female BMMCs, and two were upregulated in male BMMCs [51]. Two notable genes involved in granule formation and vacuole acidification, *Lyst* and *Slc18a2*, were downregulated in females [52, 53].

**Table 2:** Expression of genes in female BMMCs relative to male BMMCs, involving granule biogenesis and maturation

Gene name	Gene symbol	Comments	Statistics	
			Log_2_ fold change	*q* value
Spermidine synthetase	*Srm*	Synthesizes polyamines important in granule biogenesis and homeostasis, including storage of histamine and proteases (Garcia-Faroldi, 2010 [47])	3.68	0.00016
Ornithine decarboxylase 1	*Odc1*	Synthesizes polyamines important in granule biogenesis and homeostasis, including storage of histamine and proteases (Garcia-Faroldi, 2010 [47])	2.52	0.00016
Spermine synthetase	*Sms*	Synthesizes polyamines important in granule biogenesis and homesostasis, including storage of histamine and proteases (Garcia-Faroldi, 2010 [47])	0.54	0.0045
Basic helix-loop-helix family, member A15	*Bhlha15*	Important transcription factor in forming mature secretory granules in a variety of specialized secretory cells (Tian 2010 [72])	3.42	0.00016
Rab5c, member of Ras oncogene family	*Rab5c*	Key regulator of mast cell granule fusion during biogenesis (Azouz 2014 [48])	0.62	0.00032
Mannose-6-phosphate receptor	*M6pr*	Transports glycosylated proteins, such as lysosomal hydrolases and TNF, into vesicles (Coutinho 2012 [50], Olszewski 2006 [73])	0.43	0.00016
Adaptor protein 1A complex	*Ap1m1*	Protein necessary for the sorting of proteins into vesicles and secretory granule maturation (Bonnemaison 2014 [49])	0.36	0.0033
ATPase, H+ transporting, lysosomal 16 kDa, V0 subunit c	*Atp6v0c*	Component of a vacuolar type H+ ATPase that acidifies granule lumen for condensation of granule contents (Borges 2011)	0.69	0.00016
ATPase, H+ transporting, lysosomal 50/57 kDa, V1 subunit H	*Atp6v1h*	Component of a vacuolar type H+ ATPase that acidifies granule lumen for condensation of granule contents (Borges 2011)	0.52	0.00016
ATPase, H+ transporting, lysosomal V0 subunit a2	*Atp6v0a2*	Component of a vacuolar type H+ ATPase that acidifies granule lumen for condensation of granule contents (Borges 2011)	0.46	0.00016
ATPase, H+ transporting, lysosomal 50/57 kDa, V1 subunit H	*Atp6v1a*	Component of a vacuolar type H+ ATPase that acidifies granule lumen for condensation of granule contents (Borges 2011)	0.42	0.020
ATPase, H+ transporting, lysosomal 9 kDa, V0 subunit e1	*Atp6v0e*	Component of a vacuolar type H+ ATPase that acidifies granule lumen for condensation of granule contents (Borges 2011)	0.32	0.0044
ATPase, H+ transporting, lysosomal 34 kDa, V1 subunit D	*Atp6v1d*	Component of a vacuolar type H+ ATPase that acidifies granule lumen for condensation of granule contents (Borges 2011)	−0.24	0.038
ATPase, H+ transporting, lysosomal 13 kDa, V1 subunit G2	*Atp6v1g2*	Component of a vacuolar type H+ ATPase that acidifies granule lumen for condensation of granule contents (Borges 2011)	−1.84	0.00016
Lysosomal trafficking regulator	*Lyst*	Important regulator of granule formation (Durchfort 2012 [95], Hammel 2010 [52])	−1.29	0.00016
Solute carrier family 18, member 2	Slc18a2	Transports histamine and serotonin into granules (Merickel 1995 [96])	−0.92	0.00016

Our data demonstrating higher protein levels of histamine, tryptase, and chymase in female BMMCs, along with increased mRNA expression in female BMMCs for *Tnf* and *Tph1* genes with qRT-PCR (Additional file 4: Figure S4 A-B), agree with the differential gene expression discovered through RNA sequencing. Overall, these findings indicate that MCs derived from females have increased expression of many genes involved in granule biogenesis and maturation compared to MCs from males. The differences in gene expression may explain the formation of granules with increased mediator content found in female MCs.

## Discussion

The incidence, onset, and severity of many allergic, inflammatory diseases, and functional bowel diseases in which the MC is a central perpetrator of pathology are known to exhibit a sex difference. The mechanisms underlying sex-related differences in these diseases remain poorly understood. Here, we demonstrate that MCs from males and female mice exhibit distinct and vast biological differences related to their transcriptome and synthesis and storage of MC granule mediators. These differences were associated with increased release of MC mediators and pathophysiological responses to allergic and psychological stress-induced disease in females.

In the present study, female mice exhibited markedly higher IgE-mediated serum histamine responses and more severe anaphylaxis (clinical scores and hypothermia) compared with male mice. Furthermore, we demonstrated that heightened MC responses and associated pathophysiology in female mice were not restricted to IgE-dependent models of PSA, as female mice exposed to a psychological RS exhibited greater serum histamine responses and intestinal permeability without differences in corticosterone secretions between the sexes. Findings from the RS model have particular relevance to highly prevalent functional GI disorders such as IBS, for which psychological stress is a major risk factor; females are two to four times more likely than males to develop IBS [10, 54, 55].

Sex is increasingly understood to be an important variable influencing disease, and more research models are investigating these sex differences [11, 56]. Previous studies addressing the influence of sex on MC responses have focused primarily on the effects of sex hormones, particularly the direct effects of estrogen on in vitro MC degranulation [18–24]. These studies showed that estrogen signaling could enhance degranulation in MCs, indicating a role for sex hormones in promoting MC degranulation. Hox et al. [57] demonstrated that female C57BL/6 mice developed more severe hypothermia in response to PSA [57], which supports our findings in the present study. The authors also showed that ovariectomy reduced the magnitude of hypothermia; however, ovariectomized female mice in their study were not directly compared with a male cohort in that experiment and thus could not conclude that estrogen alone was responsible for the sex differences observed. In contrast to our findings presented here, Hox et al. [57] reported that, despite the increased hypothermia observed in female mice in their study, there were no differences in serum histamine responses between males and females in PSA. One key difference that likely explains the differences between our results and that from Hox et al. [57] is that they measured serum histamine 90 s after inducing anaphylaxis, whereas we measured serum histamine 30 min after inducing anaphylaxis, when serum histamine levels have been shown to reach their peak values [58].

In the present study, we demonstrated that primary MCs derived from female mice and rats possessed an increased capacity for MC mediator synthesis and granule storage compared with males. This resulted in an increased amount of MC granule mediator release as demonstrated by higher levels of β-hexosaminidase, TNF-α, tryptase, and histamine upon FcεR1 crosslinking and A12387-induced MC degranulation. As reported above, previously published work suggests a role for the direct stimulatory effects of estrogen on MC degranulation in culture. The findings from the current study are different from previous studies as we describe increased ability of female stem-cell derived MCs to synthesize and store more mediators in the absence of exogenous estrogen. Thus, the sexually dimorphic effects on MC phenotype can be assumed to be predetermined. Our findings are supported by a previous study showing that pMCs from female rats had higher histamine levels than pMCs from male rats (275.0 and 46.61 fluorescence intensity units, respectively), but this was not statistically significant, possibly due to a large standard deviation in the data [59]. Our experiments in the present study also confirmed that the elevated MC mediator release from female MCs was not associated with increased upstream p-tyrosine signaling pathways or downstream elevated intracellular Ca^2+^ mobilization. These findings also corroborated with our findings that female MCs exhibited higher release of MC mediators in response to A21387, a Ca^2+^ ionophore that degranulates MCs predominantly via downstream influx of extracellular Ca^2+^. Overall, these new findings are significant because they represent a new mechanism, other than direct stimulatory effects of sex hormones, which may contribute to the increased prevalence and severity of MC-mediated diseases in females.

There is epidemiological evidence that the estrous cycle, characterized by fluctuations in sex hormones, namely estrogen and progesterone, influences the severity of MC-associated disease in females [60–62]. Studies in rodents have shown that in multiple tissues, MC presence and staining properties can be influenced by stage of estrous cycle [37–39]. In the present study, serum histamine levels after RS and histamine content in pMCS were independent of estrous cycle stage, while slightly higher pMC numbers were found during proestrus and estrus in female mice. This suggests that the sexually dimorphic responses seen in MC disease pathophysiology were not dependent solely on the effects of sex hormones, but instead, other factors must be involved in programming the sexually dimorphic phenotype of the MC.

RNA sequencing analysis in BMMCs derived from sexually mature female and male mice revealed that, at baseline conditions (unstimulated), there were over 8000 differentially expressed genes. Specifically, our analysis revealed upregulation of multiple genes associated with the formation of immune mediators stored in preformed granules, thus supporting our cell culture and in vivo data. Sexually dimorphic gene expression has been demonstrated in a wide variety of organisms from worms to primates [63]. A study by Yang et al. [64] found that mouse liver, adipose, and muscle tissue all had tissue-specific sexually dimorphic gene expression, in which thousands of genes were differentially expressed between the sexes. Many studies have also demonstrated a large number of differentially expressed genes between the sexes in human tissues, such as peripheral blood, skeletal muscle, T cells, and jejunum [40, 65–67]. Therefore, although sex differences at the DNA level are restricted to the sex chromosomes, transcription of DNA differs widely between the sexes across many species and in many tissues. Of particular relevance to our in vivo and in vitro MC culture experiments was that the phosphoribosyl pyrophosphate synthetase enzymes were upregulated in female BMMCs. These enzymes are crucial to synthesize histidine and tryptophan, which are then converted to the preformed immune mediators histamine and serotonin, found in mast cell granules [44, 68]. Interestingly, the genes *prps1* and *prps2* map to the X chromosome, and the rate of transcription of *prps1* in particular determines the rate of production for phosphoribosyl phosphate, which in turn can be converted to histidine and tryptophan [69]. The increased biosynthesis of these amino acids may lead to an increased stock of product to be converted by histidine decarboxylase and tryptophan hydrolase to histamine and serotonin that are stored in mast cell granules. Therefore, although the gene for histidine decarboxylase, an important enzyme in histamine synthesis and granule maturation, was not upregulated in female BMMCs in the present study; the increased histamine storage in female BMMCs may be a result of the larger stores of histidine to be converted into histamine [70]. In addition, the gene for tryptophan hydrolase, the enzyme that converts tryptophan to serotonin, was upregulated in female BMMCs, suggesting that female MCs may contain higher levels of serotonin through increased stores of tryptophan and more enzyme for conversion. The gene for TNF-α was one of the most differentially expressed genes between male and female BMMCs, which correlated with our finding that female BMMCs release more TNF-α upon stimulation than male BMMCs. We also discovered increased gene expression of multiple chymases in female BMMCs as well as increased chymase protein activity. Surprisingly, a gene for tryptase was downregulated in female BMMCs even though female BMMCs had greater tryptase activity. However, post-transcriptional regulatory pathways influence functional protein abundance, which may explain our findings [42]. Numerous cathepsins were upregulated in female BMMCs and many of these cysteine proteases are upstream activators of the enzymatically inactive pro-tryptase to the mature form of tryptase, which may be another factor for the higher level of active tryptase found in female BMMCs [43]. Taken together, the sequencing results here demonstrate sexual dimorphism in MC gene expression which correlate with our in vivo and in vitro experiments.

Another interesting finding is the higher gene expression in female BMMCs than male BMMCs of the three main enzymes involved in polyamine synthesis. Polyamines are small polycations found in all mammalian cells that are important for a variety of cellular functions including chromatin condensation, DNA replication, transcription, translation, and protein activation [71]. Polyamines have a unique role in MCs because they are stored in MC granules and also necessary for dense core formation and histamine storage in MC granules [47] which also confirming our findings of increased histamine content in female MCs.

A gene that was also highly upregulated in female mast cells is *Bhlha15*, which is also referred to as *Mist1*. MIST1 is a transcription factor associated with cells that have high secretory activity, such as immunoglobulin secreting cells, alveolar breast lobular cells, and gastric zymogenic cells [72]. *Mist1*-null mice were shown to have small, underdeveloped secretory vesicles in zymogenic chief cells, demonstrating the importance of this transcription factor in vesicle formation and maturation, which may explain our findings in female MCs [72]. The mannose-6-phosphate pathway has been implicated in sorting glycosylated proteins with an M6P group (lysosomal hydrolases and TNF-α) into lysosomes [50, 73]. The mannose-6-phosphate receptor gene was upregulated in female MCs, which potentially allows increased transport of TNF-α and acid hydrolases (e.g., β-hexosaminidase) into female MC granules. The adaptor protein 1A complex (AP-1A), encoded by *Ap1m1*, is known to facilitate protein entry into vesicles, and blocking AP-1A led to the formation of immature secretory granules in AtT-20 corticotrope tumor cells [49]. The higher expression of this gene in female BMMCs compared to male BMMCs could potentially influence the increased storage of female MC granules. Five genes involving the vacuolar type H+ ATPase (V-ATPase) were upregulated in females, and two were downregulated. V-ATPase is involved in acidifying granule lumens to condense the contents and, therefore, may be involved in increasing the amount of mediators stored in granules [51].

Upon pathways analysis, a particularly interesting finding is that female BMMCs have higher expression of genes involved in metabolic pathways, including many of the biosynthetic and energy-generating pathways. Female BMMCs also had higher gene expression in cellular component organization and biogenesis than male BMMCs, including ribosome biosynthesis. These findings correlate with the ability of female MCs to be more actively producing cells that store and release more immune mediators than male MCs.

Regardless, there may be other factors involved in the transcriptomic and phenotypic differences found between male and female MCs in our study. One possibility is that sex could be influencing the epigenetic code of the cells causing sex-specific chromatin remodeling and, therefore, regulating which genes are expressed. A previous study demonstrated that sex is an important factor in DNA methylation status, which is important in altering chromatin structure and gene expression [74]. Estrogen can influence methylation patterns, and it is possible that, in the stem cells we collected, sex hormones caused epigenetic changes that had long-lasting effects on genome expression and stem cell development into MCs [75, 76]. The complement of sex chromosomes in a cell can also influence genome-wide DNA methylation in a hormone-independent fashion, demonstrating that sex hormones are not the only variable involved in the sexual dimorphism seen in MCs [77].

The X chromosome contains approximately 1100 genes, many of which are involved in immunity [78]. Females inherit two X chromosomes, one of which is randomly silenced during X chromosome inactivation, but previous studies showed up to 7% of genes escape silencing from the inactive X chromosome in mouse tissues [79] and 15% in human cells [80]. The effect of gene escape from X chromosome inactivation is not known, but extra expression of genes potentially involved in immunity may have large consequences on the development and activity of the cell.

In summary, the data presented here demonstrate the sexual dimorphism that exists in the transcriptome and biology of MCs and in the pathophysiologic responses to immunological and psychological stressors. Our data here demonstrate that female rodent MCs possessed an increased capacity to synthesize and store granule mediators, contributing to the elevated release of MC granule mediators and associated downstream pathophysiological and clinical responses. These new findings are likely to have important implications for diseases such as allergy/anaphylaxis and IBS, where the prevalence and disease severity is greater in females.

## Conclusions

The present study demonstrates that MCs exhibit marked, sexually dimorphic responses in that female MCs exhibit increased synthesis, storage, and release of MC granule-associated mediators that is independent of the estrous cycle. Differences observed in female MCs translated to a heightened tissue MC degranulation response and more severe systemic and intestinal disease activity in murine models of immunological and psychological stress. Together, these data provide a new understanding of the influence of biological sex on MC biology and disease. Further elucidation of the mechanisms driving sexual dimorphism in MCs could result in new sex-specific therapeutic targets for stress-related MC disorders exhibiting a female predominance including IBS and allergy.

## Additional files

Additional file 1: Figure S1. Basal release of histamine and morphological appearance of female and male BMMCs. (a) Female BMMCs released 36.0 ng/10^6^ cells of histamine into supernatant and male BMMCs released 19.4 ng/10^6^ cells of histamine in unstimulated conditions (*P* < 0.05; *n* = 5). (b) Representative photomicrographs of male and female BMMCs showing no noticeable differences in cell morphology between the sexes in unstimulated conditions. Values represent mean ± SE. **P* < 0.05 vs. males. (PDF 135 kb)
Additional file 2: Figure S2. Cytokine mRNA expression of IgE-DNP stimulated female and male BMMCS. (a–f) Real-time quantitative PCR of *Tnf*, *Il6*, *Il1β*, *Il4*, *Il13*, and *Ccl3* mRNA transcripts from IgE-DNP stimulated male and female BMMCs at 0, 1, 2, and 4 h normalized to *Hrpt* and relative to 0 h (*n* = 3). Values represent mean ± SE. **P* < 0.05. (PDF 83 kb)
Additional file 3: Figure S3. Sex steroid receptor gene expression in female and male BMMCs. (a–f) Real-time quantitative PCR of *Esr1*, *Esr2*, *Gper1*, *Ar*, *Pgr*, *Pgrmc2* mRNA transcripts from unstimulated male and female BMMCs normalized to *Hrpt* (*n* = 3). Values represent mean ± SE. **P* < 0.05, ***P* < 0.01 vs. males. (PDF 69 kb)
Additional file 4: Figure S4.
*Tnf* and *Tph1* gene expression in female and male BMMCs. (a) Real-time quantitative PCR of *Tnf* mRNA transcripts from 6 week-old male and female BMMCs normalized to *Rpl4* (*n* = 3). (b) Real-time quantitative PCR of *Tph1* mRNA transcripts from 6 week-old male and female BMMCs normalized to *Rpl4* (*n* = 3). Values represent mean ± SE. †*P* < 0.10, **P* < 0.05 vs. males. (PDF 102 kb)

## Abbreviations

BMMC: Bone marrow-derived mast cells
DNP-HSA: 2,4-Dinitrophenyl-human serum albumin
FD4: 4 kDa fluorescein isothiocyanate dextran
i.p.: Intraperitoneal
IBS: Irritable bowel syndrome
MC: Mast cell
pMCs: Peritoneal mast cells
PSA: Passive systemic anaphylaxis
RS: Restraint stress
TEM: Transmission electron microscopy
