# Supplementary figures and images for: Sexual dimorphism in the mast cell transcriptome and the pathophysiological responses to immunological and psychological stress
Source: Biol Sex Differ. 2016 Nov 22;7:60. doi: 10.1186/s13293-016-0113-7 (PMC5120457; doi:10.1186/s13293-016-0113-7)

**FIGURE S1: Basal release of histamine and morphological appearance of female and male BMMCs.**

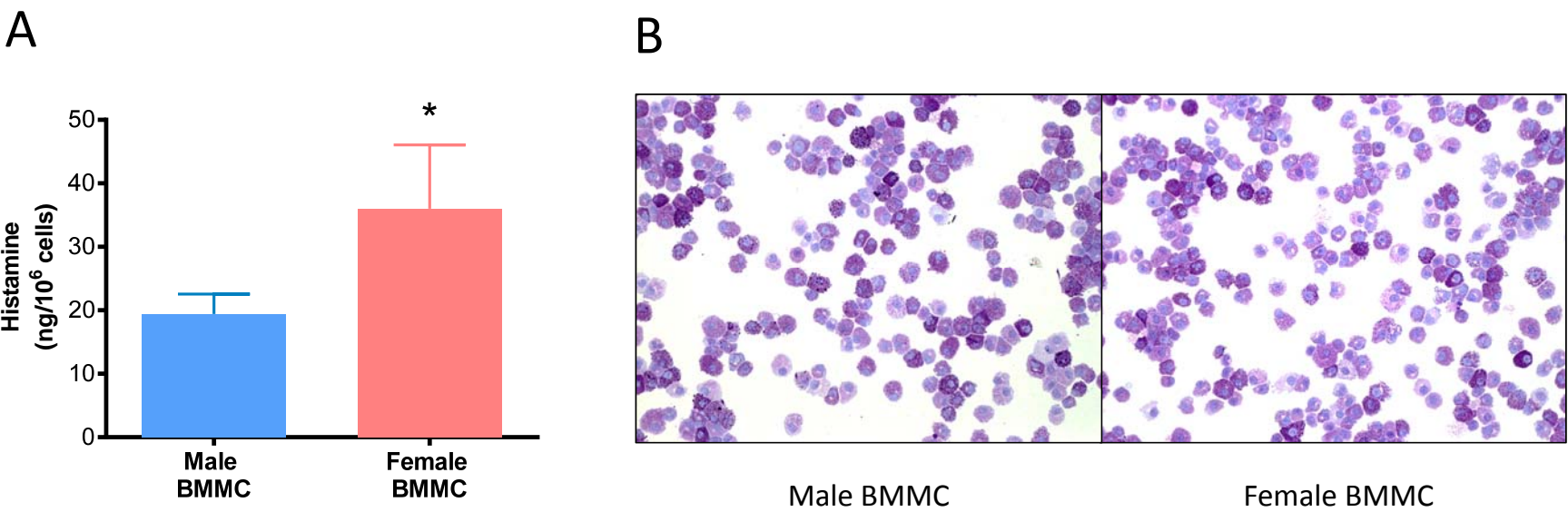

Supplement: Additional file 1: Figure S1. — Basal release of histamine and morphological appearance of female and male BMMCs. (a) Female BMMCs released 36.0 ng/106 cells of histamine into supernatant and male BMMCs released 19.4 ng/106 cells of histamine in unstimulated conditions (P < 0.05; n = 5). (b) Representative photomicrographs of male and female BMMCs showing no noticeable differences in cell morphology between the sexes in unstimulated conditions. Values represent mean ± SE. *P < 0.05 vs. males. (PDF 135 kb) [file 13293_2016_113_MOESM1_ESM.pdf]

FIGURE S2: Cytokine mRNA expression of IgE-DNP stimulated female and male BMMCS.

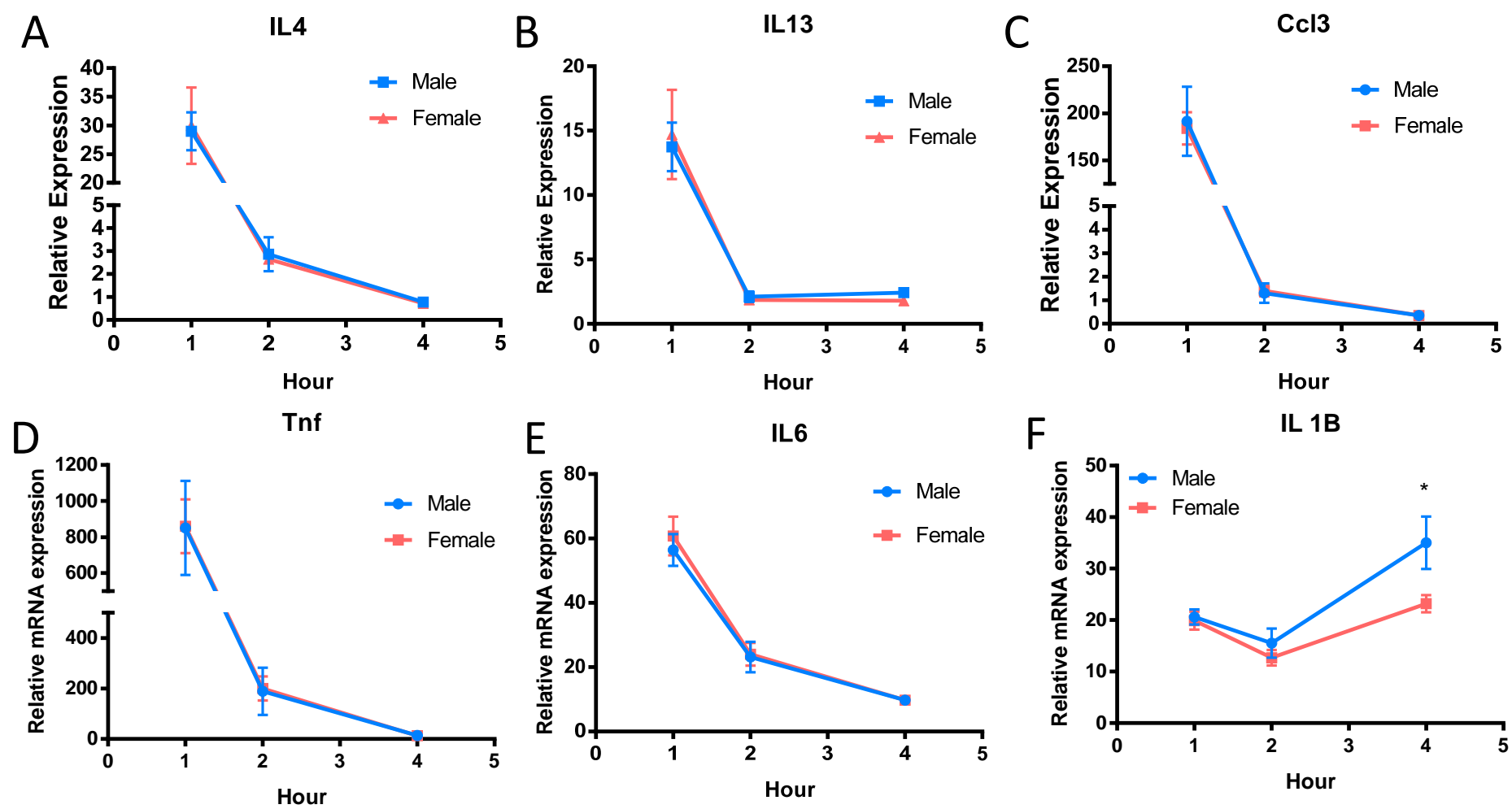

Supplement: Additional file 2: Figure S2. — Cytokine mRNA expression of IgE-DNP stimulated female and male BMMCS. (a–f) Real-time quantitative PCR of Tnf, Il6, Il1β, Il4, Il13, and Ccl3 mRNA transcripts from IgE-DNP stimulated male and female BMMCs at 0, 1, 2, and 4 h normalized to Hrpt and relative to 0 h (n = 3). Values represent mean ± SE. *P < 0.05. (PDF 83 kb) [file 13293_2016_113_MOESM2_ESM.pdf]

**Figure S3: Sex steroid receptor gene expression in female and male BMMCs.**

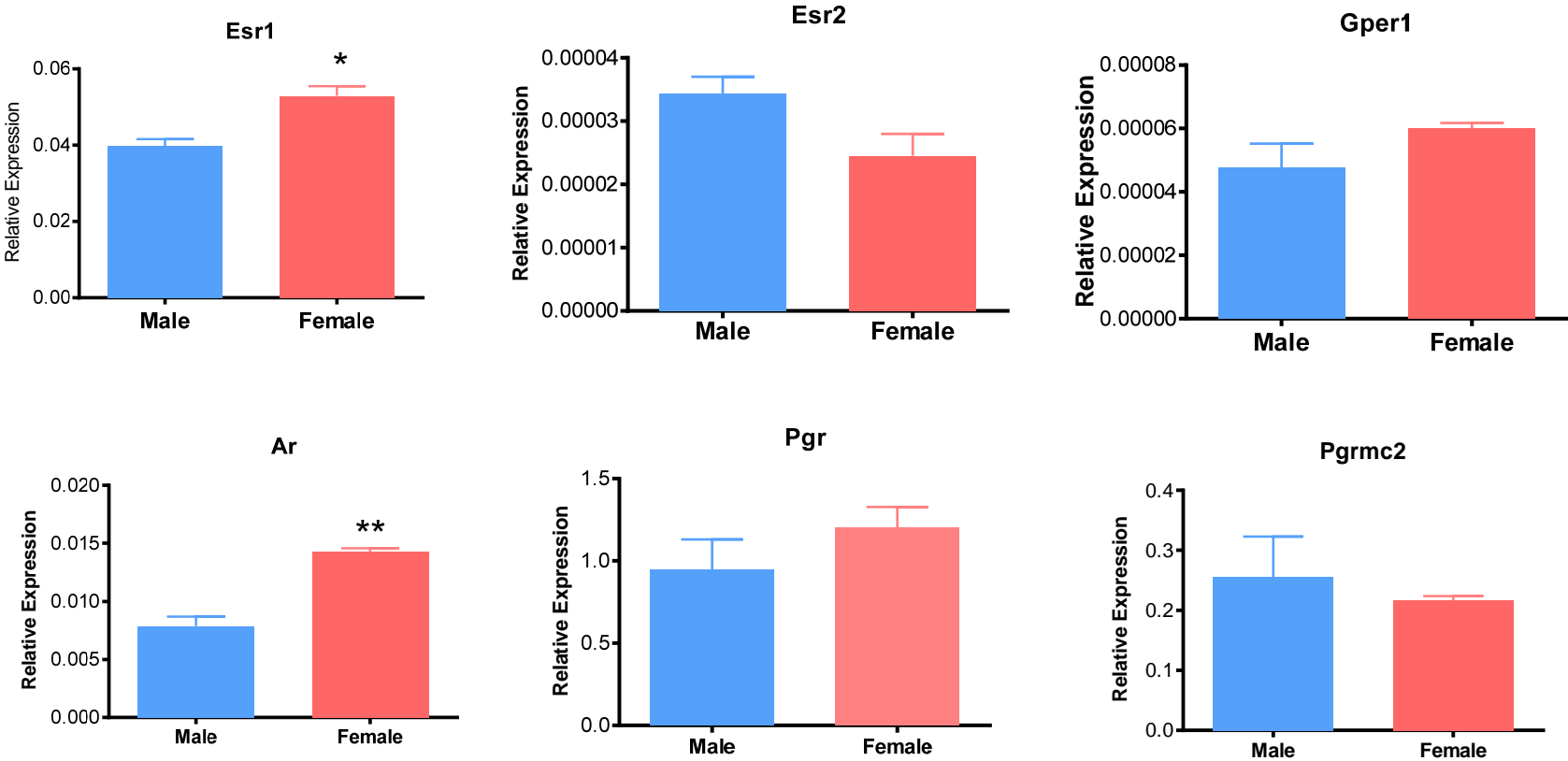

Supplement: Additional file 3: Figure S3. — Sex steroid receptor gene expression in female and male BMMCs. (a–f) Real-time quantitative PCR of Esr1, Esr2, Gper1, Ar, Pgr, Pgrmc2 mRNA transcripts from unstimulated male and female BMMCs normalized to Hrpt (n = 3). Values represent mean ± SE. *P < 0.05, **P < 0.01 vs. males. (PDF 69 kb) [file 13293_2016_113_MOESM3_ESM.pdf]

Figure S4: *Tnf* and *Tph1* gene expression in female and male BMMCs.

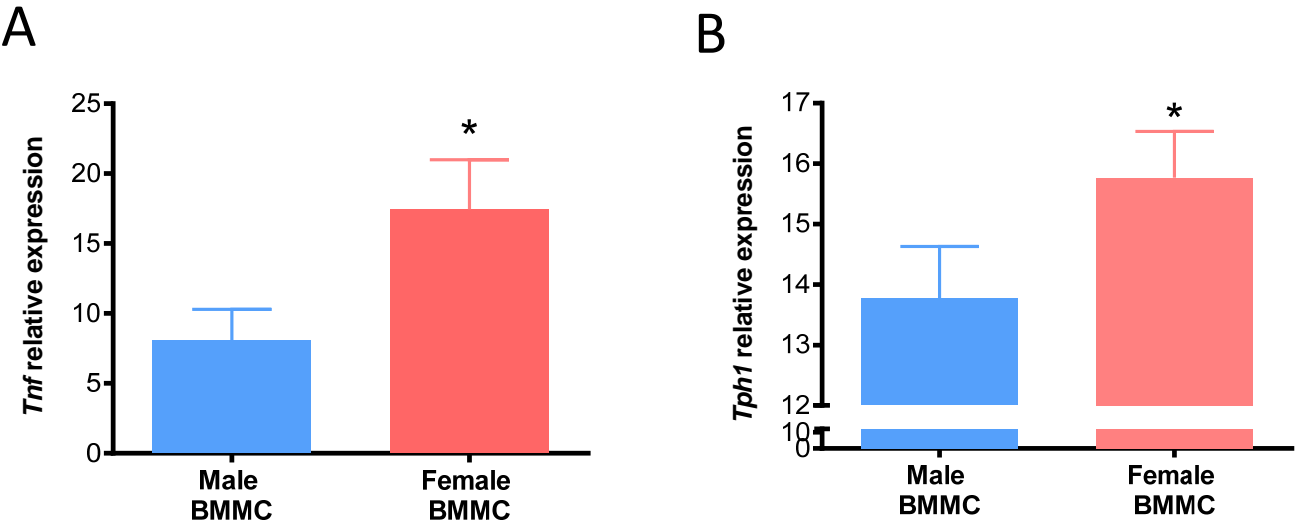

Supplement: Additional file 4: Figure S4. — Tnf and Tph1 gene expression in female and male BMMCs. (a) Real-time quantitative PCR of Tnf mRNA transcripts from 6 week-old male and female BMMCs normalized to Rpl4 (n = 3). (b) Real-time quantitative PCR of Tph1 mRNA transcripts from 6 week-old male and female BMMCs normalized to Rpl4 (n = 3). Values represent mean ± SE. †P < 0.10, *P < 0.05 vs. males. (PDF 102 kb) [file 13293_2016_113_MOESM4_ESM.pdf]
